# Retinal and Choroidal Thickness in Patients with High Myopia without Maculopathy

**DOI:** 10.12669/pjms.336.13726

**Published:** 2017

**Authors:** Kuddusi Teberik, Murat Kaya

**Affiliations:** 1 Kuddusi Teberik, Assistant Professor, Department of Ophthalmology, Duzce University Medical School, Duzce, Turkey; 2 Prof. Murat Kaya, MD, Department of Ophthalmology, Duzce University Medical School, Duzce, Turkey

**Keywords:** Axial length, Choroidal thickness, High myopia, Retinal thickness, Spectral-domain optical coherence tomography

## Abstract

**Objective:**

To evaluate macular choroidal thickness (CT) and retinal thickness in Turkish patients with high myopia without maculopathy and in normal subjects and to examine the association with age, axial length (AL), best corrected visual acuity (BCVA), cycloplegic refraction, and spherical equivalent (SE).

**Methods:**

This prospective study was performed between January 2015 and June 2016 in the Department of Ophthalmology, Duzce University Medical Faculty. It had 65 individuals (30 patients with high myopia, 35 healthy subjects). Retinal and choroidal images were obtained using spectral-domain optical coherence tomography (OCT). Measurements (one subfoveal, three temporal, three nasal) were taken at 500 μm intervals up to 1,500 μm using the caliper system. Only the right eye was used for subsequent analysis.

**Results:**

The mean age was 29.5 ± 14.5 years and 25.6 ± 7.0 in the high myopia and control groups, respectively. The subfoveal CT was significantly lower in the high myopia group (mean, 218.3 ± 102.25 mm) than the control group (mean, 331.83 ± 99.06 mm; p < 0.001). In both groups, the choroid was thinnest at the nasal 1,500 μm location, being 158.40 ± 90.8 μm and 301 ± 103.59 μm, respectively. Retinal thickness in both groups was thickest at the nasal 1,500 μm location and thinnest in the subfoveal region. In patients with high myopia, CT was negatively correlated with AL (r=-0.490, p=0.006) and age (r=-0.455, p=0.012).

**Conclusions:**

Highly myopic eyes have a thinner choroid, which may be secondary to longer AL but is not an independent factor. Further studies in the field of OCT are important to exploring the pathology of high myopia.

## INTRODUCTION

Myopia is an eye condition in which light coming through the pupil focuses not directly on the retina but rather in front of it. Myopia is one of the most common eye conditions, the leading cause of visual impairment, and a significant source of social and economic burden; however, the mechanism behind it remains unknown.^1^ In young Asian adults (ages 18–24 years), the prevalence of myopia (spherical equivalent [SE] < –0.50 diopters [D]) and high myopia (SE > –6.00 D) is 81.6–96.5% and 6.8–21.6%, respectively.^2^,^3^ However, the incidence of high myopia varies by ethnic group and country.

High myopia is associated with multiple eye morbidities. The term *high myopia* is used to refer to eyes with greater than –6.00 D refractive error.^4^ Hospital- and population-based investigations have shown that optic disc and parapapillary atrophy start to enlarge at about –8.00 D of refractive error or an axial length (AL) of ~26.5 mm. Beyond these values, the prevalence of myopic retinopathy and glaucomatous optic neuropathy increases.^5^ High myopia is characterized by excessive and progressive elongation of the globe, resulting in a variety of secondary fundus changes that may lead to visual impairment, including retinal detachment, myopic macular schisis, macular hole, choroidal neovascularization, and zonal areas of chorioretinal atrophy.^6^

In highly myopic eyes, the earliest changes begin in the choroid; thus, recent studies have focused on the choroid as an important structure involved in the pathophysiology of high myopia.^7^ The outer retina (including the photoreceptors) is nourished by the choroidal vasculature, and extreme choroidal thinning and loss of vascular tissue often lead to photoreceptor damage and visual dysfunction.^8^ Choroidal thickness (CT) may be an important parameter in studying the pathogenesis of high myopia. Accurately measuring CT *in vivo* is an essential step in monitoring disease onset and progression, which leads to choroidal thinning.

Spectral-domain optical coherence tomography (SD-OCT) is a noninvasive, noncontact, transpupillary imaging modality for investigating retinal structure. Its high signal-to-noise ratio and high scanning speed provide images of higher resolution and contrast than conventional time-domain OCT. Newly developed software for SD-OCT that uses an 830 nm infrared light source has increased our ability to image the choroid. Since Spaide et al. described enhanced depth imaging (EDI)-OCT, CT has been studied in many chorioretinal diseases using this technology.^9^ Several studies have found that in highly myopic eyes, CT is significantly thinner than in normal eyes. However, the majority of these studies did not adjust for potential compounding factors, such as AL and SE, which have a negative correlation with CT.^10^

We evaluated macular CT and retinal thickness in Turkish patients with high myopia without maculopathy and in normal subjects and examined their association with age, AL, best corrected visual acuity (BCVA), cycloplegic refraction, and SE.

## METHODS

This prospective study was performed with 65 individuals (30 patients with high myopia, 35 healthy subjects) between January 2015 and June 2016 in the Department of Ophthalmology, Duzce University Medical Faculty. The study was approved by the Institutional Ethics Committee (Clinical Trial Protocol number: 2014/40) and conducted in line with the principles of the Helsinki Declaration. All participants provided written informed consent before any study-related procedure.

High myopia was defined as SE > –6.00 D and AL ≥26 mm. Consecutive patients were included in the study. Patients were imaged as part of routine clinical ophthalmic care. To explore the presentation of high myopia without complications or myopic maculopathy, lacquer cracks, and posterior staphyloma that can change the choroidal morphology, any instances of amblyopia, eyes with a history of retinal detachment, scleral buckling, pars plana vitrectomy, photodynamic therapy, antivascular endothelial growth factor therapy, macular hole, epiretinal membrane, vitreomacular traction syndrome, foveoschisis, diabetes, any type of glaucoma, or posterior uveitis were excluded. The control group consisted of healthy individuals who visited the ophthalmology clinic for reasons only refractive errors and who had healthy eyes with SE between ±2 D.

### Ophthalmological evaluation

Demographic data from those involved in the study were obtained. After refraction measurements were performed, BCVA was tested in all cases using the Snellen chart. Snellen visual acuities were converted to logarithm of the minimum angle of resolution (logMAR) by line scoring for statistical analysis. Intraocular pressure was measured via the Goldman applanation tonometry (Nikon, Tokyo, Japan). Subjects underwent slit-lamp examination. Binocular indirect ophthalmoscopy was performed approximately 30 minutes after topical instillation of three drops tropicamide and 2.5% phenylephrine administered 5 minutes apart. Dilated fundus examination was performed by ophthalmologists. Three measurements of AL were made with Echoscan US 500. (Nidek Co. Ltd., Aichi, Japan). The average of 3 measurments was recorded.

Retinal and choroidal images of SD-OCT were obtained using an Heidelberg Spectralis (version 1.5.12.0; Heidelberg Engineering, Heidelberg, Germany). In addition to taking conventional OCT scans, we imaged the choroid with the Heidelberg Spectralis by positioning the instrument close enough to the eye to obtain an inverted image, which is referred to as enhanced depth imaging OCT. Sections, each composed of 100 average scans generated using eye tracking, were obtained in a 5 × 30° rectangle encompassing the macula and optic nerve. Using the Heidelberg Spectralis, the horizontal section going directly through the center of the fovea was used to measure

1) Central foveal thickness, defined as the distance between the inner border of the hyperreflective line corresponding to the internal limiting membrane and the inner border of the hyperreflective line corresponding to the retinal pigment epithelium.

2) CT, measured from the outer surface of the hyperreflective line ascribed to the retinal pigment epithelium to the hyperreflective line of the inner sclera border.^9^

Diurnal variation was observed in choroidal thickness measurements, as reported by Tan et al. in their study of 12 healthy volunteers.^11^ All SD-OCT images were obtained between 9.00 and 11.00 AM. Measurements (one subfoveal, three temporal, three nasal) were obtained at 500 μm intervals up to 1,500 μm using the caliper system. Spectralis OCT does not allow AL to be input, and our methods may have had residual error (2-7%) due to ocular magnification from methods that also use AL. For each subject, only the right eye was chosen for subsequent analysis. All patients under went SD-OCT measurment by a single ophthalmologist (KT). Retinal thickness measurement is automatically measured by SD-OCT. However, choroidal thickness measurements were performed by other ophthalmologist (MK) who did not have the clinical information of the patients.

### Statistical analysis

Statistical analyses were performed using SPSS for Windows (Version 17; SPSS Inc., Chicago, IL, USA). Normal distributions of continuous variables were measured by Kolmogorov - Smirnov test. Categorical variables were compared using a chi-square test. The significance of differences between mean values of the groups was evaluated using Independent sample t test, whereas the significance of the difference in median values was evaluated using the Mann–Whitney U-test. Pearson’s correlation analyses were used to measure the strength and direction of the linear relationship between two variables. A p value of <0.05 was considered statistically significant.

## RESULTS

The high myopia group consisted of 30 eyes from 22 women and eight men. The control group consisted of 35 eyes from 35 healthy patients. The mean ± standard deviation age was 29.5 ± 14.5 years (range, 13–66 years) and 25.6 ± 7.0 (range, 19–39 years) in the high myopia and control groups, respectively. The study groups were comparable in terms of sex (p=0.92) and age (p=0.52; Table-I).

**Table-I:** Demographic and clinical characteristics of study subjects.

	*High myopia (n=30)*	*Control (n=35)*	*p value*
Age (years)	29.5 ± 14.5 (24, 13-66)	25.5 ± 7.1 (23, 19-39)	0.47
Gender (male/female) n (%)	8 (27%) / 22 (73%)	9 (26%) / 26 (74%)	0.93*
Settlement (rural/urban) n (%)	11 (37%) / 19 (63%)	13 (37%) / 22 (63%)	0.96*
Cycloplegic refraction (D)	-8.5 ± 4.5	-0.3 ± 0.69	<0.001
SE (D)	-9.6 ± 2.92 (-8.88, -18 - -8)	-0.3 ± 0.68 (-0.25, -2 - 1)	<0.001
AL (mm)	27.5 ± 1.2	23.3 ± 1.01	<0.001
BCVA (logMAR)	0.25 ± 0.26	0	<0.001

D: diopter; SE: spherical equivalent; AL: axial length; BCVA: best corrected visual acuity. Data are presented as n/n or mean ±standard deviation.

* Chi-square test.

Cycloplegic refraction, SE, AL and BCVA showed significant differences between the high myopia and control groups (Table-I). Mean subfoveal CT was significantly lower in the high myopia group (mean, 218.3 ± 102.25 μm; range, 63–435 μm) than in the control group (mean, 331.83 ± 99.06 µm; range, 108–549 μm; p < 0.001). AL was 27.5±1.2 mm (range, 26.00–32.1 mm) in the high myopia group, significantly higher than in the control group (mean, 23.3 ± 1.01 mm; range, 21.3–25.6 mm; p < 0.001). The mean SE was –9.6 ± 2.92 D in the high myopia group and –0.3 ± 0.68 D in the control group (p < 0.001). BCVA on the Snellen chart was 0.25 ± 0.26 and 0.0 according to logMAR in the high myopia and control groups, respectively (p < 0.001).

### Choroidal and retinal thicknesses at different locations

In the high myopia group, the mean subfoveal choroidal thickness (SFCT) was 218.83 ± 102.25 μm and the mean macular retinal thickness was 222.23 ± 26.79 μm (Table-II). The choroid was significantly thinner in the high myopia group than in the control group (p < 0.001). In the high myopia group, the choroid was thinnest at the nasal 1500 μm location, followed by the nasal 1000, nasal 500, and temporal 500 μm locations (p < 0.001 for all). In comparison, the choroid in the control group was thinnest at the nasal 1500 μm, followed by the temporal 1500, nasal 1000, and nasal 500 μm locations (p < 0.001 for all). However, in both the high myopia and control groups, the choroid was thinnest at the nasal 1,500 μm location, being 158.40 ± 90.8 μm and 301 ± 103.59 μm, respectively (Table-II). Retinal thickness in both the high myopia and control groups was thickest at the nasal 1,500 μm location and thinnest in the subfoveal region.

**Table-II:** Distribution of choroidal and retinal thickness at different locations across the high myopic and control groups.

*Location*	*Choroidal thickness (µm)*			*Retinal thickness (µm)*		

	*High myopia (n=30)*	*Control (n=35)*	*p value*	*High myopia (n=30)*	*Control (n=35)*	*p value*
Subfoveal	218.8±102.2	331.8±99.0	<0.001	222.2±26.7	224.4±11.7	0.67
Temporal 500	221.1±94.2	330.1±92.4	<0.001	291.5±27.0	289.6±21.9	0.758
Temporal 1000	225.4±92.6	325.6±87.6	<0.001	311.4±37.4	330.5±19.0	0.015
Temporal 1500	223.0±99.7	315.4±82.2	<0.001	293.9±51.9	326.6±19.6	0.002
Nasal 500	201.9±92.1	319±101.2	<0.001	292.0±31.5	294.7±21.8	0.728
Nasal 1000	180.0±88.9	318.7±99.2	<0.001	271.9±59.2	342.2±27.4	<0.001
Nasal 1500	158.4±90.8	301±103.5	<0.001	312.8±53.8	352.1±13.6	<0.001

Data are presented as mean ± standard deviation.

### Correlation between clinical parameters and SFCT

In patients with high myopia, SFCT was negatively correlated with AL (r=-0.490, p=0.006) and age (r=-0.455, p=0.012; Table-III). However, there was weak negative correlation between AL and SFCT in the control group (r=-0.237, p=0.170). There was a negative correlation between BCVA according to logMAR and SFCT (r=-0,171 p=0.365), a positive correlation between cycloplegic refraction and SFCT (r=0.424, p=0.019), and a positive correlation between SE and SFCT (r=0.581, p=0.001) in the high myopia group. In the control group, cycloplegia refraction and SE were not significantly correlated with SFCT (p=0.427 and p=0.529, respectively). When we considered the SFCT and AL of subjects in the high myopia and control groups, we found that for each millimeter of AL, SFCT decreased by 34.87 μm in eyes with high myopia. A 12.04 μm decrease in mean SFCT was observed for each myopic diopter increase.

**Table-III:** Correlation coefficients (r) showing the linear relationship between subfoveal choroidal thickness and clinical parameters in the high myopic and control groups.

	*High myopia (n=30)*		*Control (n=35)*	

	*r*	*p*	*r*	*p*
Age	-0,455	0.012	-0.055	0.752
AL	-0.490	0.006	-0.237	0.170
BCVA^a^(logMAR)	-0.171	0.365	-	-
Cycloplegic refraction	0.424	0.019	0.139	0.427
SE	0.581	0.001	0.110	0.529

AL: axial length; BCVA: best corrected visual acuity; SE: spherical equivalent.

a BCVA of control group could not be computed because at least one of the variables is constant.

## DISCUSSION

In this study, we observed differences between high myopia and control eyes with respect to choroidal thickness, AL, cycloplegic refraction, SE, and BCVA. CT was significantly lower in the high myopia group than the control group at any location.

The mean SFCT in the high myopia group was 218.83 ± 102.25 μm, which was thicker than that reported in previous study, showing a mean CT of 123.74 ± 30.90 μm.^12^ The mean SFCT in the control group was 331.83 ± 99.06 μm, which was thicker than previously reported mean thicknesses of 272–315 μm.^13^,^14^ However, Gupta et al. found an average SFCT of 375.15 μm in emmetropes.^15^ The differences in CT in our study compared to other studies may be due to differences in participant characteristics such as age, refractive error, or ethnicity. Our study participants were young (mean age, 29.5 years) compared to participants in other studies.^12^,^16^ In addition, the differences in findings may be due to differences in software, OCT light source, ethnicity, or patient profiles (such as refractive error, eye tracking method, or AL). According to our results, CT decreased with an increase in the severity of myopic refractive error.

We observed a significant negative association between SFCT and AL, which is not surprising. In highly myopic eyes, excessive axial elongation of the eyeballs can cause biomechanical stretching and thinning of the choroid, retina, and sclera.^17^,^18^ Ikuno et al. showed significant associations between choroidal thickness, SE, CT, and posterior staphyloma height but not AL.^19^ The tessellated group in a study by Wang et al. showed no correlation between AL/SE and CT, despite the fact that the diffuse chorioretinal atrophy group (with higher AL and SE) showed strong correlations with both variables.^13^ Takahashi et al.^20^ observed a correlation between CT and AL, and Chen et al.^21^ found one between CT and SE.

In our study, there was a negative correlation between SFCT and age, AL, SE, and cycloplegic refraction but a positive correlation with BCVA. Flores-Moreno et al.^16^ reported that the relationship between age and CT in patients with high myopia was similar to that of a control group after they accounted for the effects of AL. They also suggested that AL has the strongest effect on CT in highly myopic eyes (r=-0.740). However, SFCT was strongly associated with myopic refractive error and age, as described previously.^22^ In a study by Shao et al., better BCVA was associated with thicker SFCT after the researchers adjusted for age, AL, and other parameters. However, that study was a population-based study, with a mean participant age of 64.3 years and a mean refractive error (SE) of –0.18 D.^23^ Flores-Moreno et al. examined 60 eyes of 46 highly myopic subjects (SE ≥ –6.00 D; mean age, 45.9 years) without macular diseases and found that in univariate analyses, BCVA (logMAR) was significantly correlated with thinner SFCT (r=-0.36, p=0.004).^16^ Ho et al.^24^ evaluated 56 myopic subjects (SE ≥ –6.00 D; mean age, 50.4 years) and found that in univariate analyses, SFCT was correlated with log MAR VA (r=0.295, p=0.008). The mean age of participants in our study was 29.5 years, and there was a positive relationship with SFCT because we used BCVA according to the Snellen chart. However, Gupta et al. reported no significant relationship between decreased visual acuity and a thin choroid in a subgroup of 105 patients with high myopia with or without maculopathy.^15^

In our study, there was a significant difference between retinal thicknesses at distances of 1,000 μm and 1,500 μm from the fovea, in both the nasal and temporal regions, but no difference was found at other measurement sites. Although retinal thickness was more reduced in myopic eyes (mean thickness, 285.12 μm) than in control eyes (mean thickness, 308.63 μm), a thinning of 26.51 μm may not be clinically meaningful. The photoreceptor layer may not have been disrupted, maintaining fairly normal visual function in adolescents and young adults with high myopia compared to their aged counterparts, in which the severity of both myopia and other myopic maculopathy increases, leading to decreased VA. Gupta et al. observed a significant difference in retinal thickness between myopic and emmetropic groups in all retinal quadrants, the thickest being in the nasal location and the thinnest in the subfoveal region.^15^

We observed a 12.04 μm decrease in mean SFCT for each myopia diopter increase in our study. Fujiwara et al. showed a strong correlation between CT and SE, with a decrease of 8.7 μm per negative D.^22^ Similarly, Flores-Moreno et al. found a reduction of 9.39 μm per negative D.^16^

### Limitations of the study

First, CT measurements were performed manually because automated software was not available in our clinic. Second, the majority of study subjects were female; some of our findings may not generalize to males because previous studies have observed higher CT in men than women after adjusting for age and AL.^25^ Third, because we did not account for AL to correct for ocular magnification, there may have been some residual error. Finally, this study included a nonconsecutive series with a small sample size. However, EDI-OCT was used in previous study to measure CT with reproducibility and reliability.^24^

## CONCLUSION

Highly myopic eyes have a thinner choroid, which may be secondary to longer AL but is not an independent factor. Longitudinal studies are important to increasing understanding of the pathology of high myopia, but further developments in the field of OCT are required for these studies to be most effective.
